# MAXPEEM: a spectromicroscopy beamline at MAX IV laboratory

**DOI:** 10.1107/S160057752300019X

**Published:** 2023-02-03

**Authors:** Yuran Niu, Nikolay Vinogradov, Alexei Preobrajenski, Claudia Struzzi, Brice Sarpi, Lin Zhu, Evangelos Golias, Alexei Zakharov

**Affiliations:** a MAX IV Laboratory, Lund University, Box 118, 22100 Lund, Sweden; ESRF – The European Synchrotron, France

**Keywords:** X-ray photoelectron microscopy, ACLEEM, micro-ARPES, XMCD

## Abstract

MAXPEEM, a dedicated photoemission electron microscopy beamline at MAX IV Laboratory, houses a state-of-the-art aberration-corrected spectroscopic photoemission and low-energy electron microscope (AC-SPELEEM). This powerful instrument offers a wide range of complementary techniques providing structural, chemical and magnetic sensitivities with a single-digit nanometre spatial resolution. The beamline can deliver a high photon flux of ≥10^15^ photons s^−1^ (0.1% bandwidth)^−1^ in the range 30–1200 eV with full control of the polarization from an elliptically polarized undulator. The microscope has several features which make it unique from similar instruments. The X-rays from the synchrotron pass through the first beam separator and impinge the surface at normal incidence. The microscope is equipped with an energy analyzer and an aberration corrector which improves both the resolution and the transmission compared with standard microscopes. A new fiber-coupled CMOS camera features an improved modulation transfer function, dynamic range and signal-to-noise ratio compared with the traditional MCP-CCD detection system.

## Abstract 2

The design and performance of a new dedicated spectromicroscopy beamline at the Swedish national synchrotron (MAX IV) is presented.

## 1. Introduction

In the last decades, photoemission combined with high spatial resolution has seen remarkable growth (Bauer, 2014 ▸). Photoemission electron microscopy (PEEM) is an important surface characterization tool. Similar to conventional optical microscopy, it employs full-field microscopy, which utilizes photo-exited electrons to form the image. Owing to major instrument development in the last few decades, especially with the incorporation of low-energy electron microscopy (LEEM), energy filters and synchrotron light sources, PEEM has become a powerful instrument that can provide various contrast mechanisms, multiple operation modes, and complementary measurement capabilities with spatial, momentum, energy and temporal resolutions. Synchrotron-based PEEM (XPEEM) has been extensively used in a wide range of disciplines, *e.g.* materials science, nano-science, heterogeneous catalysis, corrosion science, biology and biomineral science, to image structural, chemical, electronic and magnetic properties of the surface and nanostructures (Shi *et al.*, 2018 ▸; Laverock *et al.*, 2018 ▸; Ali-Löytty *et al.*, 2018 ▸; Bommanaboyena *et al.*, 2021 ▸; Rullik *et al.*, 2016 ▸; Hjort *et al.*, 2014 ▸; Fitzer *et al.*, 2016 ▸; Metzler *et al.*, 2007 ▸). To perform such measurements with single-digit nanometre resolution, one has to design a beamline with high photon density flux and install a state-of-the-art low-energy electron microscope with a high-resolution detection system. In this paper, we outline the design and characteristics of the MAXPEEM beamline which houses an aberration-corrected low-energy electron microscope and demonstrate its performances. The aberration corrector not only allows the user to improve the spatial resolution but also increases the microscope transmission. In turn, the higher transmission enables the user to decrease the total flux on the sample, thus diminishing the space-charge effect and beam damage at the surface. The MAXPEEM beamline is located at the 1.5 GeV synchrotron ring of the MAX IV laboratory, Sweden.

Many synchrotron facilities in the world have PEEM beamlines but only a few are equipped with an aberration corrector (AC). At Diamond Light Source (beamline I06; Dhesi *et al.*, 2010 ▸), an aberration-corrected low-energy electron microscope (AC-LEEM) has recently been installed but no performance data are available yet. At NSLS II (Reininger *et al.*, 2012 ▸), which hosts a similar instrument, the main difference is the spot size (50 µm) and grazing incidence illumination. At Shanghai Synchrotron Radiation Facility (Xue *et al.*, 2014 ▸), an aberration-corrected photoemission electron microscope (AC-PEEM) is used for time-resolved photoelectron microscopy (Li *et al.*, 2019 ▸). There are also two non-commercial aberration-corrected PEEM instruments: one is SMART type at BESSY II [a complete version with LEEM function and a dedicated Omega-type energy filter (Schmidt *et al.*, 1998 ▸)] and the other one is PEEM3 at the Advanced Light Source (beamline 11.0.1.1). Among all these microscopes MAXPEEM is unique due to the small photon beam spot size, which enables higher spatial resolution measurements. Another feature of the MAXPEEM beamline is the normal incidence geometry of the incoming photon beam.

## 2. Beamline overview

The MAXPEEM beamline consists of an elliptically polarizing undulator as a light source, a collimated plane-grating monochromator with corresponding focusing and refocusing optics, and aberration-corrected spectroscopic photoemission and low-energy electron microscope (AC-SPELEEM) as the endstation.

### 2.1. Insertion device

**Figure 1:**
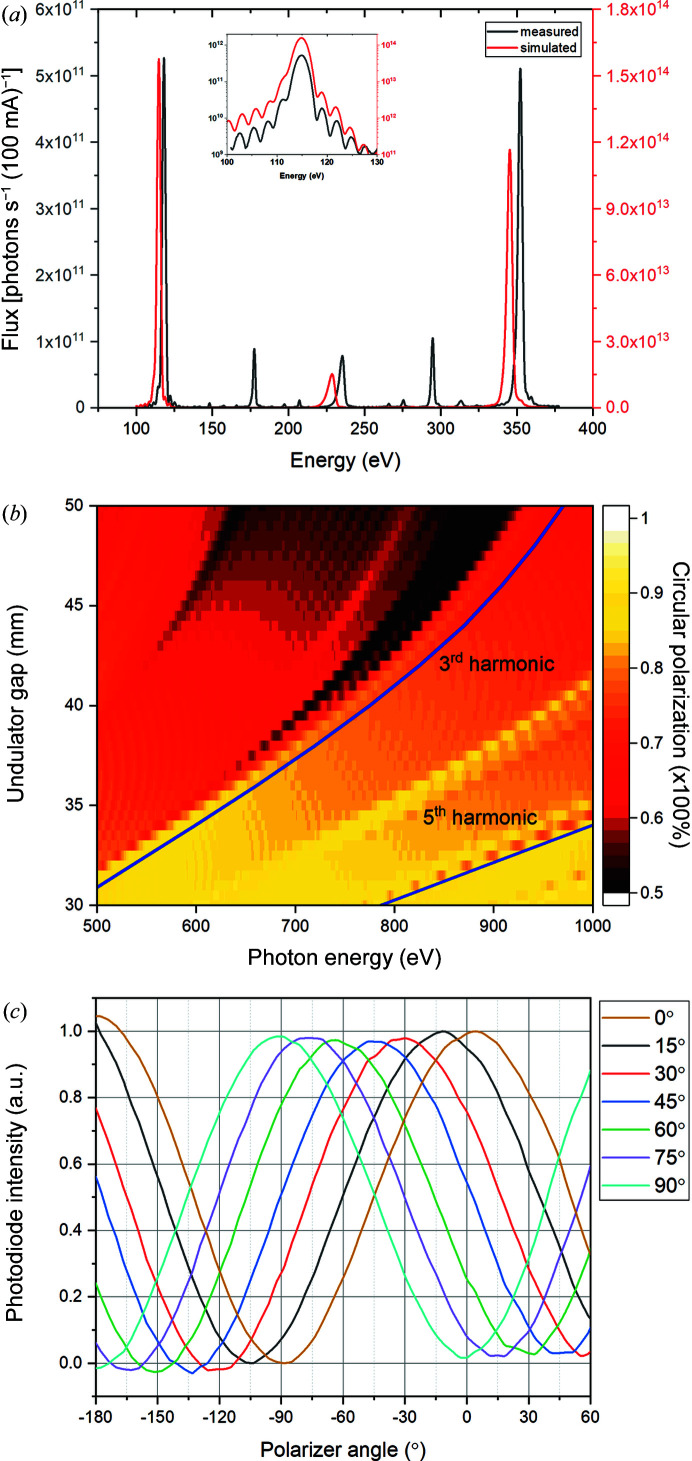
(*a*) Simulated and measured undulator spectra in planar mode for an undulator gap of 30 mm. The beamline opening is 0.1 mrad. The monochromator grating features 1221 lines mm^−1^. The inset shows the agreement between the simulated and measured spectra for the first harmonics at 115 eV (logarithmic scale on the intensity axis); the small energy shift has been corrected. The experimental undulator spectrum was measured right after the exit slit of the beamline. (*b*) Calculated map of the undulator spectra showing the degree of circular polarization of the photon beam at the fixed phase of 14 mm. The locations of the third and fifth harmonics are indicated by the blue curves. (*c*) Reflected X-ray intensities versus azimuth angles of the multilayer analyzer in the polarimeter when the undulator, *i.e.* the gap and phase, were configured to several simulated polarization angles in the inclined mode. The photon energy is 708 eV, and 0° (90°) means the polarization of the X-rays is linearly horizontal (vertical).

The source for the MAXPEEM beamline is an elliptically polarizing undulator (EPU) of Apple-II type (Sasaki *et al.*, 1993 ▸). The insertion device is called EPU58 and has 42 periods and a period length of 58 mm. The maximum radiated power is 1.46 kW (planar phase, 500 mA ring current). The estimated maximum flux into the beamline is ≥10^15^ photons s^−1^ (0.1% bandwidth)^−1^. The undulator was first tuned and characterized on a magnetic bench prior to installation on the ring. Under normal operation, the undulator demonstrates that its performance is in very good agreement with simulations [Fig. 1 ▸(*a*)] except for a small energy shift attributed to the imperfection in the mutual alignment of the insertion device and the beamline (Tanaka & Kitamura, 2001 ▸). The measured undulator spectrum of the first harmonics effectively reproduces interference fringes [inset, Fig. 1 ▸(*a*)]. The fundamental harmonics (linearly polarized light) cover the photon energy range 30–350 eV. When the EPU58 is tuned to produce circularly polarized light only the first harmonic has intensity on the optical axis and covers the photon energy range 40–300 eV. For higher harmonics, as shown in Fig. 1 ▸(*b*), the undulator must be tuned to produce elliptically polarized light and the degree of circular polarization will be less than 100%. The undulator also works in the inclined mode, in which it is possible to produce linearly polarized light at an arbitrary azimuth angle, *i.e.* from −90° to +90°. The degree of polarization in the helical mode as well as the linear polarization direction in the inclined mode have been characterized by a multilayer-based soft X-ray polarimeter (Grizolli *et al.*, 2016 ▸). For example, Fig. 1 ▸(*c*) shows that, in the inclined mode, as the multilayer analyzer rotates around the optical axis, the X-ray intensity follows a sine function nicely. The measured angles of the polarization are consistent with the simulated angles.

### 2.2. Optical layout

**Figure 2:**
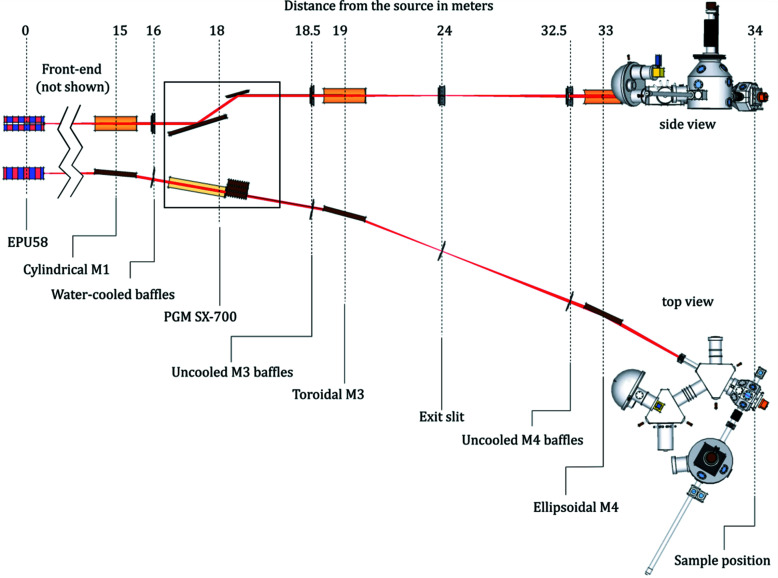
Optical layout of the MAXPEEM beamline with a single refocusing ellipsoidal mirror (M4). Distances are given in metres (not to scale).

The optical layout of the MAXPEEM beamline is shown schematically in Fig. 2 ▸. The first optical element in the beamline is a cylindrical mirror (M1) at 15000 mm after the EPU source. The mirror collimates the beam vertically before the monochromator. The size of M1 determines the acceptance of the beamline, which is selected to be 0.80 mrad (H) × 1.36 mrad (V). Horizontal acceptance is chosen to be relatively large, 8σ (σ = 0.1 mrad is the standard deviation of the angular opening of the first undulator harmonic at the lowest photon energy of 30 eV). For accepting 99.7% of the monochromatic radiation a fan of 6σ would be sufficient, but even larger horizontal acceptance makes the thermally induced deformation smoother in the central area, which is irradiated by the light of interest. The need for full illumination of M1 determines its length to be 350 mm. The high vertical acceptance follows from the fact that it is difficult to make a mirror narrower than 20 mm, and at a distance of 15000 mm this corresponds to 1.36 mrad. The water-cooled apertures in front of the monochromator can reduce both horizontal and vertical acceptance further. The monochromator is SX-700 PGM from the beamline I311 at the old MAX laboratory; it consists of a plane mirror (M2) and three interchangeable plane gratings (PGs). The dispersed radiation from the grating is focused both vertically and horizontally at the exit slit by a toroidal focusing mirror (M3). This mirror has to accept the fan of 6σ (because heat deformations are not as important as they are for M1), setting the length of M3 to 320 mm. The final re­focusing is accomplished by a single ellipsoidal mirror (M4) deflecting the beam horizontally. The optical aperture of M4 is 260 mm × 20 mm, accepting 85% intensity at 30 eV and more at higher energies (a longer M4 would provide slightly more flux at low energies but would also increase the level of slope errors, the price, and require a larger and more expensive mirror vessel and mechanics). The image at the exit slit plane is demagnified by a factor of ten at the sample position. The grazing incidence angles for M1, M3 and M4 are all 2°.

### 2.3. Mirror units

The mirror system is designed based on an idea to improve its vibrational behavior by making the movable parts as small and as lightweight as possible, avoiding stacked motion stages, and keeping the distance between a massive support block and the movable elements as short as possible. This approach has been reached by having a small cylindrical mirror chamber held and moved by five driven and one non-driven legs in a rectangular parallel kinematic arrangement. This system is able to determine all degrees of freedom. By driving the legs separately or in a combined way all three rotations, the vertical linear motion and the horizontal transversal motion can be achieved. The horizontal longitudinal direction is fixed and not motorized. The mirror motion is fully automated and incorporated into the beamline control system.

## 3. Beamline performance

**Figure 3:**
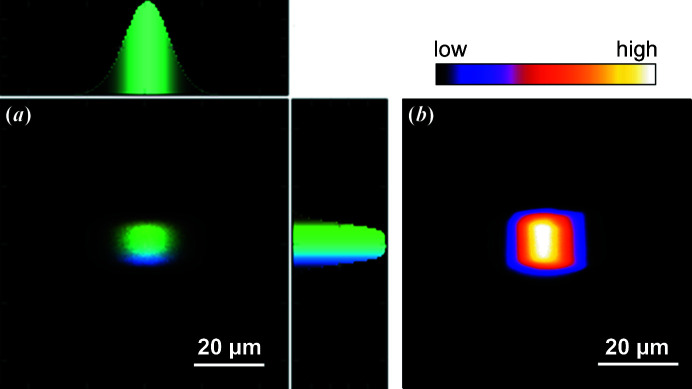
Beam profile at the sample position of the MAXPEEM beamline. (*a*) Simulation using X-ray tracing software (Klementiev & Chernikov, 2014 ▸), photon energy 40 eV; the energy slit is 150 µm (H) × 150 µm (V). The color code is the energy dispersion that scales with the photon energy. It is ±7 meV at 40 eV photon energy. (*b*) Experimental beam profile in the photoelectron microscope at 43 eV photon energy. The beamline energy slit is 150 µm × 150 µm and the signal is from secondary photoelectrons discriminated by the energy analyzer of the microscope, with an energy window of 0.2 eV.

**Figure 4:**
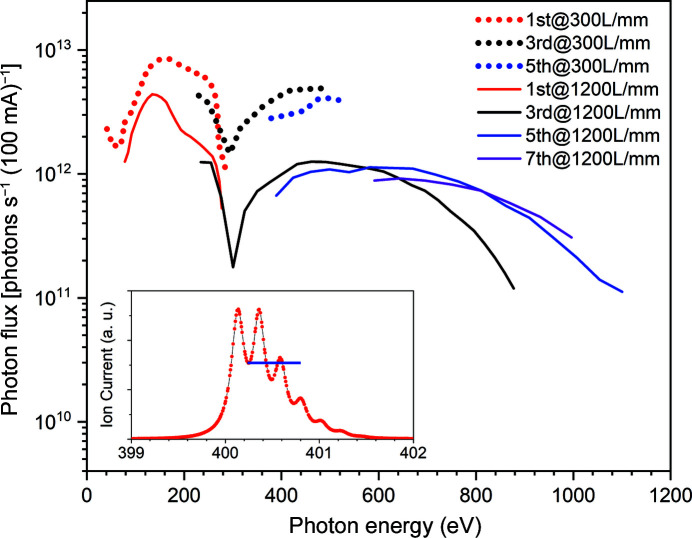
Measured photon flux after the exit slit for two different gratings, 300 lines mm^−1^ and 1221 lines mm^−1^. The beamline opening is 0.15 mrad, the energy slit is 150 µm (H) × 300 µm (V). Inset: ion yield spectrum at the N_1*s*_-absorption edge of N_2_.

The spot size at the sample position is around 15 µm (H) × 15 µm (V) (with a 0.15 mm exit slit opening vertically and horizontally) and can be reduced down to approximately 10 µm (H) × 4 µm (V) by closing the exit slit further. The relatively large horizontal spot size results from the expected tangential slope errors on M4 (0.7 arcsec r.m.s.). The spot size can also be increased up to approximately 40 µm × 40 µm by changing the yaw rotation angle of the M4 mirror, thus defocusing the photon beam. The measured and calculated optimal spot size is presented in Fig. 3 ▸ (Klementiev & Chernikov, 2014 ▸). The beamline photon flux within the main energy range 30–545 eV for the low-density (LD) grating (300 lines mm^−1^) and 70–1100 eV for the high-density (HD) grating (1221 lines mm^−1^) is shown in Fig. 4 ▸. The photon energy resolution of the beamline was measured by studying the absorption spectrum of molecular nitrogen recorded with a gas cell. The ion yield spectrum at the N_1*s*_ → 1π_g_* excitation region is shown in the inset of Fig. 4 ▸. The spectrum has been measured with the HD grating at the smallest vertical exit slit (10 µm). The instrumental broadening was estimated by calculating the intensities of the first valley in the spectrum and the third peak (Chen & Sette, 1989 ▸). A Gaussian broadening of 60 meV gives a resolving power of the monochromator of about 7000 for the HD grating. This result is consistent with its previous calibration of energy resolution carried out at the old I311 beamline from which the SX-700 monochromator was moved. The measured and calculated energy resolution in the whole photon energy range (50–1000 eV) was presented in an earlier publication (Nyholm *et al.*, 2001 ▸). The energy resolution Δ*E* changes as *E* ^3/2^ when the monochromator is operated in the fixed focus mode. The resolving power for the LD grating is 3× lower but is still sufficient for most of the measurements since it matches the energy resolution of the microscope.

## 4. Beamline and microscope control

Initially, we had three different control systems at the MAXPEEM beamline. The beamline control system (the source and the optics) is built using Tango (https://www.tango-controls.org/) and uses a Python-based Sardana framework (Coutinho *et al.*, 2011 ▸) to communicate with the IcePAP controllers (Janvier *et al.*, 2013 ▸). The microscope has its own software (*U-view2002+LEEM2000*) developed by the microscope company Elmitec GmbH (https://elmitec.de/). The camera and detector (TVIPS GmbH, https://www.tvips.com/) have standard software for electron microscopes (TVIPS GmbH). First, we incorporated the detector software into the *U-view* program and then arranged a communication of the *U-view* program with the beamline control system when steering the undulator and monochromator is needed as, for example, in the acquisition of X-ray absorption spectra.

## 5. Endstation: aberration-corrected SPELEEM microscope

**Figure 5:**
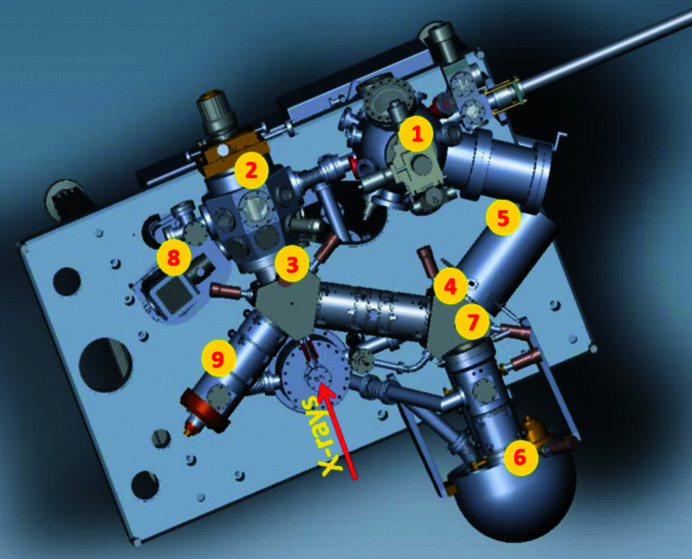
Schematic of the AC-SPELEEM endstation. (1) Preparation chamber, (2) main chamber, (3) first beam separator, (4) second beam separator, (5) AC mirror, (6) energy analyzer, (7) TVIPS-F216 detector (CMOS-camera), (8) UV lamp, and (9) electron gun (LaB_6_) and the illumination column.

The microscope combines the ability to perform XPEEM (X-ray photoemission electron microscopy), XAS (X-ray absorption microspectroscopy), small-spot XPS (X-ray photoelectron micro-spectroscopy), XPD (X-ray photoelectron diffraction, including micro-ARPES), LEEM and LEED (low-energy electron microscopy and diffraction, respectively). A schematic of the microscope is shown in Fig. 5 ▸. Component 6 in the figure is the R100 energy analyzer which was recently upgraded to R200. All images presented in the manuscript were collected with the old R100 analyzer and better performance is expected with the new energy filter. A large multi-purpose preparation and experimental ancillary chamber is attached to the microscope. It has *in situ* sample preparation facilities including material deposition, LEED measurements, gas dosing, sputtering and annealing up to 2000 K. The system is also compatible with the Universal Sample Holder (USH) system (Omicron flag-type adapter), allowing sample transfer via a Ferrovac ultrahigh vacuum (UHV) ‘suitcase’ between the microscope and other external UHV systems, *e.g.* a scanning tunneling microscope (STM) facility. The sample is hosted in the analysis chamber (or so-called main chamber) of the AC-SPELEEM microscope with a unique light incidence geometry – the photons from the synchrotron impinge the sample surface at normal incidence. This helps to avoid undesirable shadowing effects at 3D structures which is a problem for oblique-angle illumination. Another advantage of the normal incidence geometry is much better focusing in the horizontal direction, therefore, increasing the useful flux density at the sample and enabling total flux density to be lowered which then diminishes the space-charge effect. One consequence of this geometry is a potential decrease in photoionization cross-section due to the 90° angle between the polarization vector and the direction of photoelectrons (Yeh & Lindau, 1985 ▸). In the main chamber, live imaging is possible not only at varying temperatures, from 89 K to 1600 K, but also under varied external conditions, *i.e.* magnetic field, electric potential or current and material/gas deposition. In this section, we describe the microscope performance. The microscope has an aberration corrector to compensate for the spherical and chromatic aberrations of the objective lens. If the corrector is on, the second beam-splitter directs the electron beam from the intermediate imaging column into the mirror column which is a tetrode electrostatic mirror. In this way, a consistent improvement in lateral resolution and transmission is achieved. The beam separator design also allows us to switch off the mirror when the electron beam is deflected directly into the imaging column, energy analyzer and detector. The mirror-on configuration is more time-consuming and is used when higher spatial resolution and/or higher transmission is needed. Below we present the performance of the AC microscope with some application examples.

### 5.1. New fiber-coupled CMOS detector: TVIPS F216

**Figure 6:**
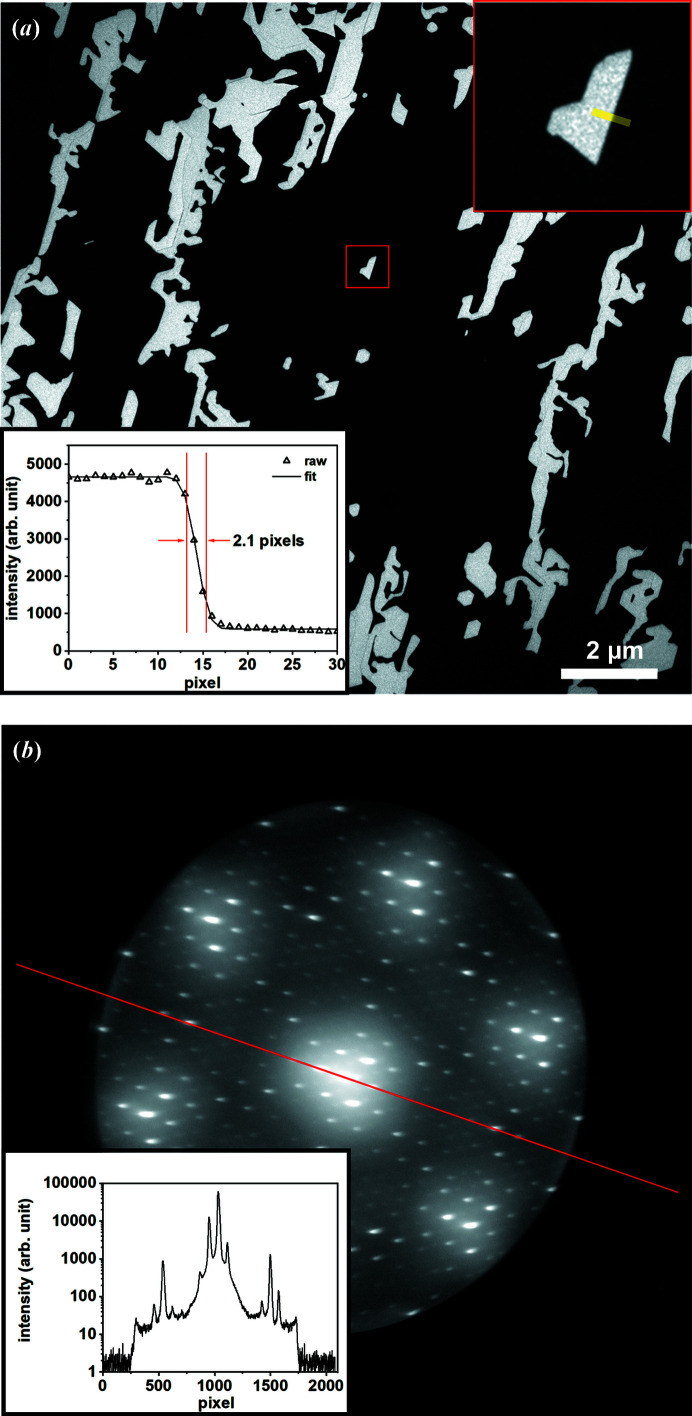
(*a*) LEEM and (*b*) micro-spot LEED images from an epitaxial mono-/bi-layer graphene sample grown on SiC to show the resolution and dynamic range of TVIPS-F216. The image size of (*a*) is 20 µm × 20 µm. The upper inset in (*a*) is the magnified image of the area selected in red (1484 nm × 1484 nm) and the profile across the mono-/bi-layer boundary (yellow line) is shown in the lower inset of (*a*). The resolution of the detector is determined to be about 2.1 pixels, which corresponds to 32.8 nm. The inset in (*b*) shows the profile along the red line across the LEED spots [note that panel (*b*) is given on a logarithmic scale].

Nowadays, most PEEM/LEEM systems use the MCP-CCD system to convert the final image from electrons into a grayscale image that can be shown and stored on a PC. However, the resolution of such a detector system is limited to about 130 µm, which corresponds to about 300 effective pixels across the 40 mm diameter of the MCP (Shimizu *et al.*, 2006 ▸; Moldovan *et al.*, 2008 ▸). The dominant mechanism of image degradation is the lateral spread of the secondary electrons in the phosphor screen. With the new generation of AC-SPELEEM microscopes, the old MCP-CCD detector will limit the overall system performance. To overcome this problem, several types of solid-state detectors that convert the electron events directly into electronic signal were once tested in PEEM/LEEM systems after many years of application in X-ray diffractometers and transmission electron microscopes (Tinti *et al.*, 2017 ▸; Tromp, 2015 ▸; van Gastel *et al.*, 2009 ▸). Recently, a more economic camera system that combines the traditional scintillator and the latest CMOS chip was adopted by several LEEM/PEEM systems (Janoschka *et al.*, 2021 ▸; see also https://www.bnl.gov/cfn/facilities/probes.php). In such systems, the 20 keV electrons first generate fluorescent photons on the scintillator layer. These photons are then transferred through the optical-fiber coupling unit to the CMOS chip. The detector is not bakeable but is very compatible with UHV as only the scintillator layer and the optical fiber array are inside the UHV chamber. After installing the detector into a pre-baked chamber that has a base pressure of 7 × 10^–11^ Torr, the chamber pressure decreases to a low −10 scale within one or two days. The detector used in our microscope (TVIPS F216 model) with 16 µm physical pixel size has demonstrated 4× higher resolution as well as two magnitudes higher dynamic range than the traditional MCP-CCD system. These improvements are shown in Fig. 6 ▸.

### 5.2. Aberrations in the Elmitec microscope

**Figure 7:**
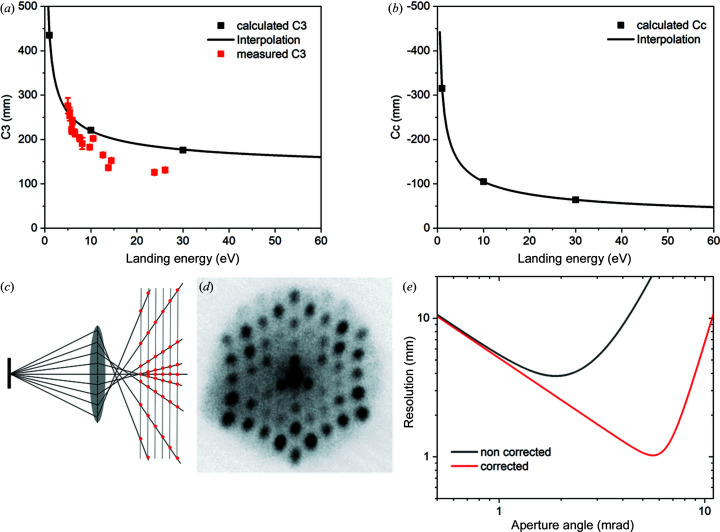
Aberration coefficients and spatial resolution in Elmitec LEEM. Panels (*a*) and (*b*) show calculated spherical (C_3_) and chromatic (C_c_) aberration coefficients at three different energies 1 eV, 10 eV and 30 eV along with the 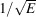 interpolation. The red squares in (*a*) are the experimentally measured C_3_ for different energies with real space micro-LEED. (*c*) Schematic of the real space micro-LEED for measuring spherical aberrations in the microscope. (*d*) Experimental real space image of the Si(111) surface, with electron energy 5.4 eV, illumination aperture 10 µm, defocus 25 mA. (*e*) Comparison of the resolution and transmission for the Elmitec microscope with and without the aberration corrector. Electron energy *E* = 10 eV, and the electron energy spread is 0.25 eV. The correction was carried out by removing only the third-order spherical and chromatic aberrations.

Correction of main aberrations (spherical and chromatic) in the electron microscope is one of the most significant breakthroughs in improving the spatial resolution of the instrument. The spherical (*C*_3_) and chromatic aberrations (*C*_c_) of the cathode lens on the image side are well understood and are given by Tromp (2011 ▸): *C*_c_ = − *L*(*E*/*E*_0_)^1/2^ + *C*_cm_ and *C*_3_ = *L*(*E*/*E*_0_)^1/2^ + *C*_3m_, where *L* is the sample–objective distance (2–3 mm) and *C*_cm_ and *C*_3 m_ are the chromatic and spherical aberrations of the magnetic part of the objective lens, respectively. *E* and *E*_0_ are the electron landing energy and the final energy after acceleration, respectively. For the Elmitec instrument, the corresponding simulations are presented as solid black dots and curves in Figs. 7 ▸(*a*) (*C*_3_) and 7 ▸(*b*) (*C*_c_). There is an elegant way to measure spherical aberrations using a micro-spot LEED with an illumination area on the order of 250–500 nm, where the image location of the diffracted beam is observed in the Gaussian image plane [so-called real space micro-LEED (Tromp, 2013 ▸)]. Fig. 7 ▸(*c*) shows a schematic of the experiment (the corrector was off), and the experimental data are presented in Figs. 7 ▸(*d*) and 7 ▸(*a*) (red dots). Using a small illumination aperture and slight sample defocusing allows us to highlight the effect of aberrations. The pattern in Fig. 7 ▸(*d*) looks like a diffraction pattern but in fact it is a real space image formed by electron beams with radial displacements that depend on the diffracted angles. The experimental spherical aberration coefficients of the microscope are in satisfactory agreement with the theoretical simulations [Fig. 7 ▸(*a*)]. With all the aberration coefficients the user can estimate the gain in the spatial resolution and the microscope transmission. From Fig. 7 ▸(*e*), based on a simple analytical calculation, the user can observe if the third-order spherical and chromatic aberrations are completely removed, we could gain 4–5× not only in spatial resolution but also in electron transmission owing to the larger size of the contrast aperture.

### 5.3. Experimental tests

#### 5.3.1. Test of the aberration corrector

**Figure 8:**
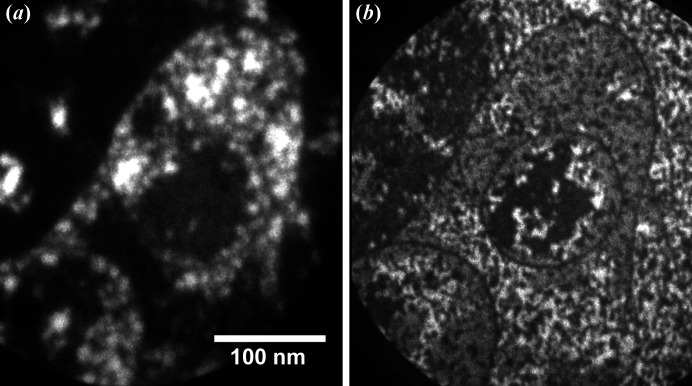
High-resolution secondary (*a*) XPEEM and (*b*) LEEM images of Sn/SnO_*x*_ intercalated graphene. For the XPEEM image, every frame was acquired with 1 s exposure time in the stack of 185 images. Before integration, the images in the stack were drift-corrected. For the LEEM image, the image was integrated from eight frames with 1 s exposure time without drift correction (note that the single step is clearly visible in both images).

**Figure 9:**
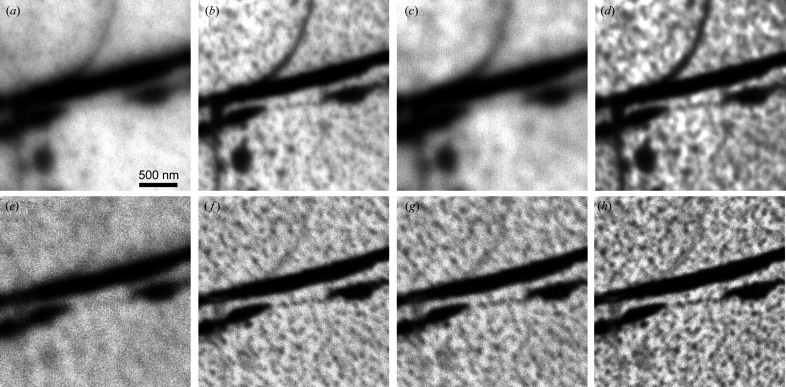
Space-charge effects manifested in the XPEEM images obtained under different conditions. Top row: secondary XPEEM images: (*a*) high photon flux of 4.2 × 10^13^ photons s^−1^, (*b*) low photon flux of 1.4 × 10^13^ photons s^−1^ in the mirror-off mode, (*c*) same as (*b*) but in the mirror-on mode, (*d*) same as (*c*) with insertion of the SAA (100 µm). Lower row: Si_2*p*_ core-level XPEEM images, (*e*) same as (*c*), (*f*) with the SAA inserted, (*g*) with the knife aperture inserted (no SAA), and (*h*) with both the SAA and the knife inserted. All XPEEM images presented are 2.5 µm × 2.5 µm, cropped from raw images with FoV = 10 µm. The sample is monolayer graphene with a few bilayer islands grown on SiC (0001). In the mirror-on (mirror-off) mode, the 70 µm (30 µm) CA was always used. The photon energy used for all images was 150 eV. The integration time for (*a*)–(*d*) was 3.2 s and (*e*)–(*h*) was 320 s.

**Figure 10:**
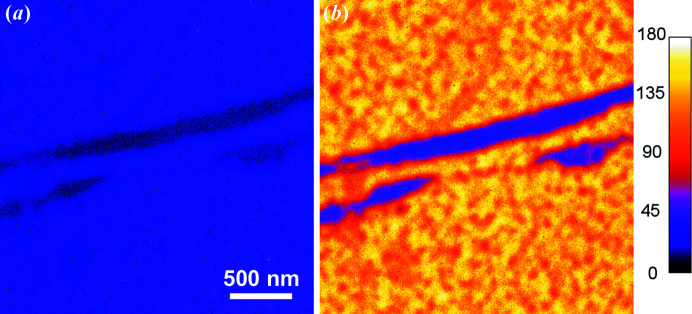
Si_2*p*_ core-level XPEEM images taken in the (*a*) mirror-off and (*b*) mirror-on modes with both the SAA and the knife inserted. For better illustration, the original gray images were converted with a fake-color map. The average intensities of the two images are 34.7 and 120.8, respectively, as different CAs were used: 30 µm in (*a*) and 70 µm in (*b*). [Note that image (*b*) is a copy of the image in Fig. 9 ▸(*h*).]

Before we discuss the performance of the aberration corrector, it is worth mentioning the spatial resolution that can be obtained without the corrector and which other factors (aside from aberrations) are crucial for the ultimate performance of the microscope. At high magnifications, a stable environment, first of all, mechanical noises, temperature and electronic stability, are the key factors for a good microscope performance. This means that a short acquisition time and subsequent drift correction of the stack of images can markedly improve the image quality. This method is particularly useful for XPEEM imaging because of low signals. For the XPEEM image, a large (several hundred) stack of images was collected at the shortest possible exposure time of the detector [*e.g.* 1 s in Fig. 8 ▸(*a*)]. Then, an image drift-correction procedure was applied to the whole stack and after that the images were integrated (Schindelin *et al.*, 2012 ▸; Tseng *et al.*, 2012 ▸). Without a corrector, the spatial resolution in the XPEEM mode is about 10 nm [Fig. 8 ▸(*a*)] whereas in LEEM mode it is about 5 nm [Fig. 8 ▸(*b*)] (Niu *et al.*, 2017 ▸). In the case of XPEEM, it is very important to keep the photon flux as low as possible to diminish the space-charge effect. Space-charge effects always occur when there is a very high electron density in the (photo)electron beam. Very often it is observed in the microscopes installed at a synchrotron (Schmidt *et al.*, 2013 ▸; Locatelli *et al.*, 2011 ▸) due to the pulse nature of the photon source. The space-charge effect first occurs at the sample surface before the acceleration and then in the electron crossovers in the imaging column. Low photon flux at the sample, *i.e.* by detuning the undulator gap, is the main factor in eliminating the space-charge effect [*e.g.* compare Figs. 9 ▸(*a*) and 9 ▸(*b*)]. In fact, in addition to the aforementioned method of image acquisition, the good resolution of Fig. 8 ▸(*a*) benefits greatly from the very careful control of space-charge effects. When the aberration-corrector is on, an extra space-charge effect occurs in the mirror column, where electrons decelerate and the space-charge effect there is even more severe than at the surface [*e.g.* compare Figs. 9 ▸(*b*) and 9 ▸(*c*)]. To diminish the space charge in the microscope, especially in the mirror column, the user has to decrease the electron beam flux inside the column. One way to do this is to introduce a field-limiting aperture (selected area aperture, SAA) during the course of image acquisition as observed in the comparison of Figs. 9 ▸(*c*) and 9 ▸(*d*) [as well as Figs. 9 ▸(*e*) and 9 ▸(*f*) with core-level photoelectrons]. The drawback of putting SAA in the XPEEM mode is image vignetting if the sampling area is less than the field of view (FoV). For example, in Fig. 9 ▸(*d*) [as well as Fig. 9 ▸(*f*)], a 100 µm SAA was inserted to reduce the space charge, which in fact reduces the visible size of the image from the original FoV of 10 µm to 5 µm. In our microscope, we introduced another remedy to mitigate the space-charge effect in the mirror column: a knife-limiting edge. This edge is installed in the middle of the intermediate imaging column, which is close to a dispersive plane of the electron beam. It cuts secondary photoelectrons thus significantly improving the quality of core-level XPEEM images [*e.g.* compare Figs. 9 ▸(*e*) and 9 ▸(*g*)]. Fig. 9 ▸(*h*) demonstrates the enhanced action of both the SAA and the knife in the aberration-corrected XPEEM imaging mode. Using the corrector and limiting the electron beam in the microscope, we can easily obtain a moderate resolution of 50 nm with much higher transmission. As shown in Fig. 10 ▸, the gain in transmission is about 3–4× and roughly scales with the opening of the contrast aperture (CA = 70 µm for the corrector on compared with 30 µm for the corrector off case). Experimentally, we observe that the photon flux has to be reduced to obtain a decent spatial resolution in both (corrector on and off) modes. The ultimate resolution with the corrector on is not necessarily better than the case when the corrector is off due to space-charge effects and other factors. Nevertheless, the gain in transmission with the corrector on can offset the signal loss quite well due to the reduction of photon flux and make the acquisition time shorter, especially when only a moderate resolution is needed.

#### 5.3.2. Micro-ARPES mode of the microscope

**Figure 11:**
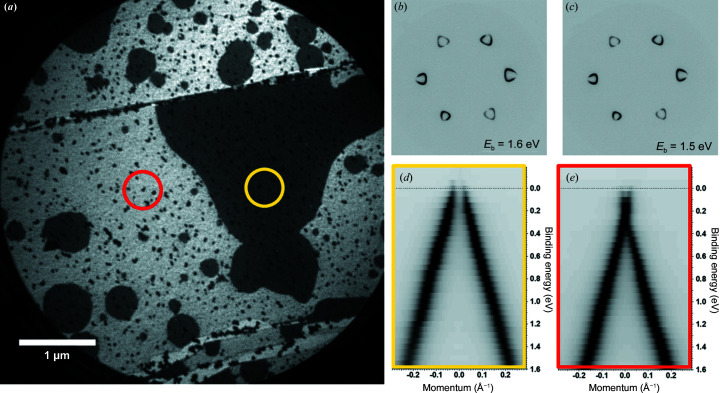
(*a*) LEEM image of Ge-intercalated graphene. The contrast in the image can be attibuted to different doping, black areas are *p*-type doped and white areas are n-type doped. On the right, selected area micro-ARPES data are shown: panels (*b*) and (*c*) are constant energy scans close to the Fermi level; panels (*d*) and (*e*) show the *E*(*k*) band structure (Dirac cone) from the marked areas (red and yellow) in (*a*). The sampling area is about 1.5 µm and the photon energy is 42.3 eV.

An X-ray photoemission electron microscope (X-PEEM) equipped with a hemispherical energy analyzer is capable of fast acquisition of momentum-resolved photoelectron angular distribution patterns in a complete cone (Zakharov *et al.*, 2011 ▸). We have applied this technique to observe the 3D (*E*, *k_x_*, *k_y_*) electronic band structure of *p*–*n* junctions in germanium intercalated graphene; the sampling area can be on a sub-micrometre scale. Fig. 11 ▸(*a*) displays a LEEM image of the surface where the contrast can be attributed to different doping (one and two monolayers of intercalated germanium underneath a free-standing graphene layer). At fixed photon energy it is possible to sweep kinetic energies and obtain an energy slice of the 2D image (*k_x_*, *k_y_*) in reciprocal space [Figs. 11 ▸(*b*) and 11 ▸(*c*)]. Using the stack of images acquired at different kinetic energies it is possible to plot ‘standard’ *E*(*k*) dependencies at different areas on the surface [marked by the red and yellow circles in the LEEM image; Figs. 11 ▸(*d*) and 11 ▸(*e*)].

#### 5.3.3. XMCD and XMLD measurements at MAXPEEM

**Figure 12:**
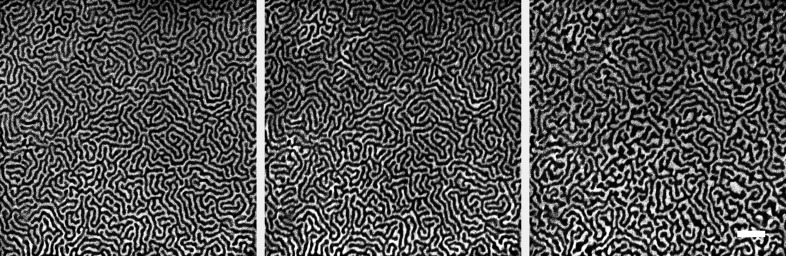
Magnetic domain patterns from XMCD measurements in a Pt /Co multilayer sample with an out-of-plane magnetic moment. Images were taken at the remanent state after applying out-of-plane magnetic fields of 0 mT (left), 25 mT (middle) and 72 mT (right). The images were taken with a photon energy of 780 eV (*L*_3_ peak in the Co XAS spectrum) and a kinetic energy of 1.6 eV. The undulator phase +(−)16 mm is used for the right (left) circular polarization. The scale bar is 1 µm.

**Figure 13:**
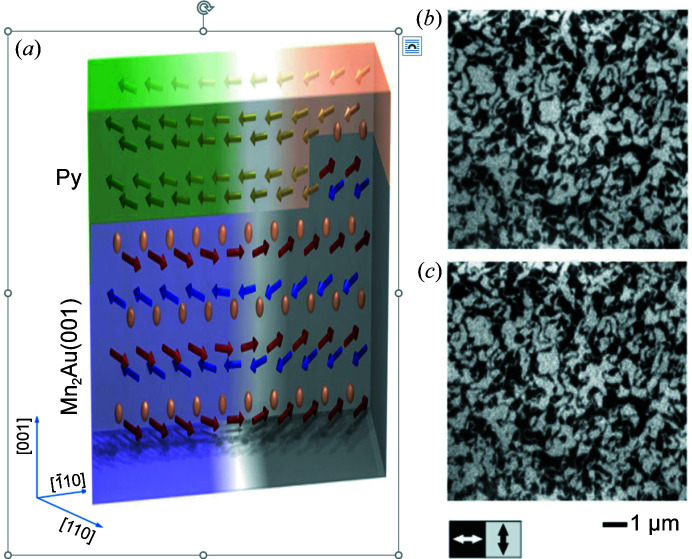
Schematic of an antiferromagnetic spintronics system of Mn_2_Au and Permalloy. The resemblance of the ferromagnetic and antiferromagnetic domain patterns is verified by XMLD images taken at the (*b*) Fe-edge and (*c*) Mn-edge, respectively. The antiferromagnetic domains of the Mn_2_Au layer can be read out by the Permalloy overlayer with strong exchange coupling between the two layers.

The normal incidence geometry of the photon beam at the MAXPEEM beamline is unique among similar beamlines around the world. It favors studies with out-of-plane magnetic moments in X-ray magnetic circular dichroism (XMCD) experiments. This is also the beamline of choice when in-plane magnetic moments in X-ray magnetic linear dichroism (XMLD) experiments are performed. Owing to the normal incidence geometry, the gain in signal is about a factor of four for XMCD, whereas, for in-plane sensitive XMLD, measurement is simplified as no sample rotation is needed. In Fig. 12 ▸ the magnetic domain patterns of a multilayer Pt/Co sample are presented. The magnetic domains with up- and down-magnetic moments were imaged by using the XMCD signal. The magnetic domain patterns can be altered by an external out-of-plane magnetic field produced with our magnetic sample holder. The photon flux of pure circular polarization mode only exists in the first harmonics which are within the energy interval 40–300 eV. In addition, the merit flux of elliptically polarized light occurs at a higher odd harmonic spectrum. The user has to optimize the flux and polarization rate on the different phasing positions of the undulator. The least photon flux of higher harmonic spectra occurs around the phasing position of 21 mm. The data in Fig. 12 ▸ were collected at a phasing position of 16 mm (*i.e.* degree of polarization ≃ 0.86) which gives an optimal tradeoff between the beamline flux and degree of circular polarization. Each XMCD image in Fig. 12 ▸ only took 80 s due to the 100% sensitivity from the normal incidence of the photon beam. With XMLD, as shown in Fig. 13 ▸, not only the antiferromagnetic domains of a Mn_2_Au film but also the ferromagnetic domains of the Permalloy layer on its top can be imaged. The resemblance of the two sets of domains demonstrates that the exchange coupling between the layers effectively copies the antiferromagnetic domain from Mn_2_Au to the ferromagnetic Permalloy layer (Bommanaboyena *et al.*, 2021 ▸). It is worth emphasizing that the normal incidence of the photon beam means neither in-plane ferromagnetic components nor out-of-plane antiferromagnetic components would be sensed in the current design of MAXPEEM. Nevertheless, the two examples above have proved the particular usefulness of MAXPEEM in XMCD and XMLD.

## 6. Conclusions

The MAXPEEM beamline is a dedicated beamline for high spatial resolution spectro-microscopy measurements. The aberration-corrected SPELEEM microscope offers a wide range of complementary techniques providing structural, chemical and magnetic sensitivity with a single-digit nanometre spatial resolution. The aberration corrector improves both the resolution and the transmission provided that the space-charge effect in the mirror column is diminished by limiting the size of the electron beam in the intermediate imaging column of the microscope. The very unique geometry of the incoming photon beam (normal incidence) implies much more effective sample illumination and the absence of shadow effects characteristic for grazing incidence geometry. In addition, it favors studies with out-of-plane magnetic moments in XMCD and in-plane magnetic moments in XMLD experiments. The beamline reaches low photon energies suitable for valence band and micro-ARPES studies. The initial user operation shows that the beamline meets the design parameters very well and performs as expected.
